# Amyloid Precursor Protein (APP) Mediated Regulation of Ganglioside Homeostasis Linking Alzheimer's Disease Pathology with Ganglioside Metabolism

**DOI:** 10.1371/journal.pone.0034095

**Published:** 2012-03-28

**Authors:** Marcus O. W. Grimm, Eva G. Zinser, Sven Grösgen, Benjamin Hundsdörfer, Tatjana L. Rothhaar, Verena K. Burg, Lars Kaestner, Thomas A. Bayer, Peter Lipp, Ulrike Müller, Heike S. Grimm, Tobias Hartmann

**Affiliations:** 1 Deutsches Institut für DemenzPrävention (DIDP), Saarland University, Homburg/Saar, Germany; 2 Neurodegeneration and Neurobiology, Saarland University, Homburg/Saar, Germany; 3 Experimental Neurology, Saarland University, Homburg/Saar, Germany; 4 Molecular Cellbiology, Saarland University, Homburg/Saar, Germany; 5 Department for Psychiatry, University of Goettingen, Goettingen, Germany; 6 Institute for Pharmacy and Molecular Biotechnology (IPMB), University of Heidelberg, Heidelberg, Germany; Boston University School of Medicine, United States of America

## Abstract

Gangliosides are important players for controlling neuronal function and are directly involved in AD pathology. They are among the most potent stimulators of Aβ production, are enriched in amyloid plaques and bind amyloid beta (Aβ). However, the molecular mechanisms linking gangliosides with AD are unknown. Here we identified the previously unknown function of the amyloid precursor protein (APP), specifically its cleavage products Aβ and the APP intracellular domain (AICD), of regulating GD3-synthase (GD3S). Since GD3S is the key enzyme converting *a*- to *b*-series gangliosides, it therefore plays a major role in controlling the levels of major brain gangliosides. This regulation occurs by two separate and additive mechanisms. The first mechanism directly targets the enzymatic activity of GD3S: Upon binding of Aβ to the ganglioside GM3, the immediate substrate of the GD3S, enzymatic turnover of GM3 by GD3S was strongly reduced. The second mechanism targets GD3S expression. APP cleavage results, in addition to Aβ release, in the release of AICD, a known candidate for gene transcriptional regulation. AICD strongly down regulated GD3S transcription and knock-in of an AICD deletion mutant of APP *in vivo*, or knock-down of Fe65 in neuroblastoma cells, was sufficient to abrogate normal GD3S functionality. Equally, knock-out of the presenilin genes, presenilin 1 and presenilin 2, essential for Aβ and AICD production, or of APP itself, increased GD3S activity and expression and consequently resulted in a major shift of *a*- to *b*-series gangliosides. In addition to GD3S regulation by APP processing, gangliosides in turn altered APP cleavage. GM3 decreased, whereas the ganglioside GD3, the GD3S product, increased Aβ production, resulting in a regulatory feedback cycle, directly linking ganglioside metabolism with APP processing and Aβ generation. A central aspect of this homeostatic control is the reduction of GD3S activity via an Aβ-GM3 complex and AICD-mediated repression of GD3S transcription.

## Introduction

**Figure 1:**
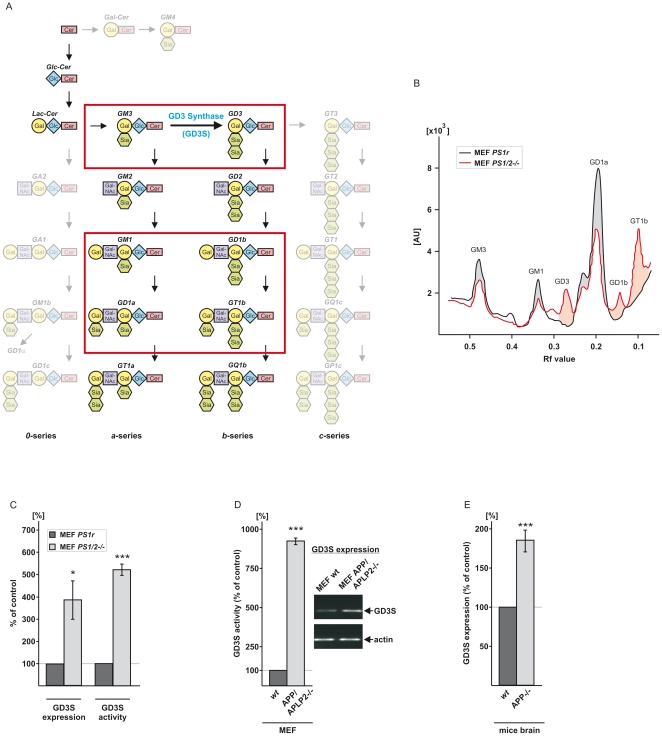
Ganglioside biosynthesis and effects on GD3s in dependence of APP-processing. (*A*) Biosynthetic pathway of ganglio-series gangliosides is shown. GD3-synthase (GD3S) converts GM3 to GD3 by adding sialic acid to GM3, generating the *b*-series of gangliosides. The major brain gangliosides GM1, GD1a, GD1b and GT1b are highlighted. (*B*) TLC analysis of *a*- and *b*-series gangliosides in PS-deficient cells. Representative densiometric quantification of TLC, analyzing the ganglioside pattern in PS1/2 deficient mouse embryonic fibroblasts (MEF PS1/2-/-) and MEF PS1/2-/- retranfected with PS1 wildtype (MEF PS1r). Peaks were identified by external standard and Rf-value. (*C*) GD3S expression and activity in PS1/2-deficient cells. In accordance with the altered *b*∶*a* ratio of gangliosides, GD3S expression and activity is increased in PS-deficient cells (MEF PS1/2-/-) compared to MEF PS1r. (*D*) Analysis of GD3S activity and GD3S expression in APP/APLP2-deficient mouse embryonic fibroblasts (MEF APP/APLP2-/-) compared to wildtype mouse embryonic fibroblasts (MEF wt). MEF APP/APLP2-/- show increased GD3S activity and enhanced corresponding GD3S expression, analyzed by quantitative real-time PCR. (*E*) *In vivo* analysis of GD3S expression in brains of APP-deficient mice (APP-/-) show increased GD3S gene transcription compared to wt mice.

Alzheimer's disease (AD) is a devastating neurodegenerative disorder, pathologically characterized by extracellular senile plaques and intracellular neurofibrillary tangles [1]. Major constituents of senile plaques are 40–42 amino acids (aa) long peptides termed β-amyloid (Aβ) [2]. Aβ is generated by sequential processing of the type-I transmembrane amyloid precursor protein (APP), involving β-secretase BACE1 [3] and γ-secretase. The β-secretase cleaves APP within the extracellular/luminal domain, generating the N-terminus of Aβ and a 99 aa long membrane-bound C-terminal fragment C99/β-CTF, which is further cleaved by γ-secretase to release Aβ and the APP intracellular domain (AICD). The γ-secretase consists of at least four proteins, presenilin 1 (PS1), presenilin 2 (PS2), nicastrin, anterior pharynx-defective 1 (Aph-1) and presenilin enhancer 2 (Pen-2) [4]. The polytopic transmembrane proteins presenilin (PS) constitute the active site of the protease [5] and mutations in the genes encoding PS1 and PS2 are responsible for most cases of familial early-onset Alzheimer's disease (EOAD) [6]. As the γ-secretase cleavage site is centered within the transmembrane domain, lipid composition of cellular membranes has been shown to influence proteolytic processing of APP [7], [8], [9], [10], [11], [12]. Beside cholesterol and sphingomyelin (SM) [8], [10], there are several strong indications which point towards an important role of gangliosides in AD pathogenesis [13], [14], [15], [16]. Gangliosides are a family of sialic acid containing glycosphingolipids, highly enriched in neuronal and glial membranes, where they play important roles for development, proliferation, differentiation and maintenance of neuronal tissues and cells [17]. The ganglioside GM3 serves as a common precursor for the *a*- and *b*-series gangliosides. The GD3-synthase (GD3S) catalyzes the synthesis of GD3 by adding sialic acid to GM3, segregating the *a*- and *b*-series of gangliosides (Fig. 1*A*) [18] and therefore controlling the levels of the major brain gangliosides GM1, GD1a, GD1b and GT1b. Gangliosides are directly involved in AD pathology, e.g. the concentration and composition of gangliosides are altered in the brains of AD patients and in transgenic mouse models of AD [19], [20], [21]; ganglioside clusters in neuronal membranes take part in the formation of amyloid fibrils [13], [15], [16]; GM1 drastically increases Aβ production [22], binds to Aβ and is discussed to act as a seed for Aβ aggregation in amyloid plaques [23], [24], [25]; and Aβ aggregation as well as Aβ induced cell death are reduced in AD-model mice lacking GD3S [14]. The underlying mechanism of how APP processing and AD interfere with ganglioside homeostasis, however, remains unclear. Here we identified a regulatory cycle in which gangliosides regulate APP processing, and Aβ and AICD control the *a*/*b*-series ganglioside homeostasis. Central to this homeostatic control is the regulation of the GD3S activity via an Aβ-GM3 complex and AICD mediated repression of GD3S transcription.

## Results

### GD3-synthase is affected in PS- and APP-deficient cells

To investigate a potential role of PS as the active part of the γ-secretase complex in ganglioside metabolism, we analyzed PS1/PS2-deficient mouse embryonic fibroblasts (MEF PS1/2-/-) [26], [27] and PS1 wild-type retransfected control cells (MEF PS1r) (Fig. S1). Lipid extraction of the major brain gangliosides [28] followed by thin-layer chromatography (TLC) analysis showed an increase in *b*-series gangliosides GD3, GD1b and GT1b in PS-deficient cells, whereas levels of the *a*-series gangliosides GM3, GM1 and GD1a were strongly decreased (Fig. 1*B*, Fig. S2). The conversion of *a*- to *b*-series gangliosides is catalyzed by the enzyme GD3S, suggesting that GD3S activity is blocked by γ-secretase activity. Indeed, quantitative real-time PCR of MEF PS1/2-/- cells showed that GD3S expression was strongly elevated and in accordance with increased GD3S expression, enzyme activity was also strongly increased in MEF PS1/2-/- cells (Fig. 1*C*), explaining the elevated *b*-series gangliosides and decreased *a*-series gangliosides observed. Beside γ-secretase cleavage of APP, γ-secretase is involved in processing numerous type-I transmembrane proteins [29]. To elucidate the mechanism how PS affects GD3S activity and to identify the γ-secretase substrate involved in GD3S activity regulation, we analyzed mouse embryonic fibroblasts devoid of APP and the APP-like protein APLP2 (MEF APP/APLP2-/-). Similar to the results obtained with PS-deficient cells, we observed an increase in GD3S activity and gene transcription in MEF APP/APLP2-/- cells (Fig. 1*D*), clearly suggesting that the cleavage of APP and/or APLP2 by PS/γ-secretase regulates ganglioside metabolism. Because PS knock-out mice are not viable, the role of PS could not be directly assessed *in vivo*. However, APP knock-out mice are viable, which allowed us to evaluate the role and *in vivo* relevance of APP in GD3S regulation directly. Indeed, the absence of APP alone increased GD3S expression in the brain (Fig. 1*E*), providing clear evidence for APP as a γ-secretase target involved in GD3S activity regulation.

### Aβ peptides decrease GD3-synthase activity by mediating substrate availability

**Figure 2:**
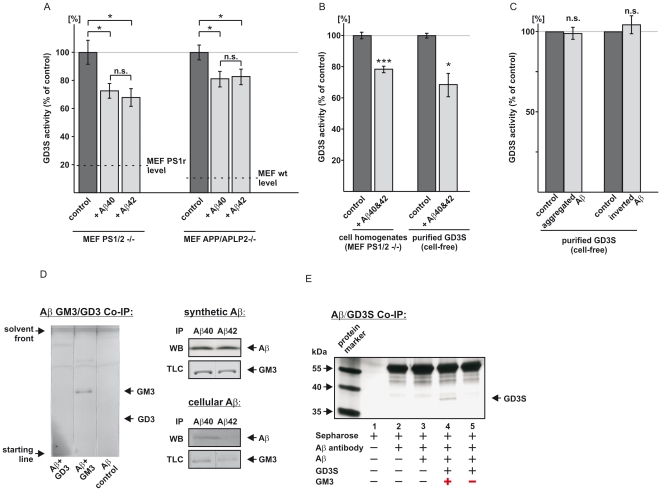
Influence of Aβ on GD3S activity. (*A*) Effect of Aβ on GD3S activity. MEF lacking Aβ because of PS- or APP-deficiency (MEF PS1/2-/- and MEF APP/APLP2-/-, respectively) were incubated with physiological Aβ40 or Aβ42 concentrations or the solvent used for the Aβ peptides (control). The level of the GD3S activity of the corresponding wildtype cells are indicated (horizontal dotted black line); corresponding wildtype/control cells for MEF PS1/2-/-: PS1/2 deficient MEF retransfected with PS1 (MEF PS1r); corresponding wildtype/control cells for MEF APP/APLP2: mouse embryonic fibroblasts of wildtype mice (MEF wt). Both Aβ species partially rescued the increased GD3S activity in PS- or APP-deficient MEF. No significant differences were observed between Aβ40 and Aβ42 peptides. (*B*) Direct effect of Aβ40 and Aβ42 on GD3S activity. Homogenates of PS1/2-/- cells incubated with Aβ40 and Aβ42 show decreased GD3S activity. Similar results are obtained with Aβ40 and Aβ42 in a cell-free assay containing only purified GD3S and the substrate GM3. (*C*) Influence of aggregated Aβ and inverted Aβ on GD3S activity. Inverted Aβ peptides and aggregated Aβ showed no influence on GD3S activity. (*D*) GM3, the substrate for GD3S, binds to Aβ: Physiological concentrations of cellular derived Aβ bind GM3 analyzed by co-immunoprecipitation (co-IP) of Aβ in presence of GD3 or GM3. GM3, but not GD3, binds to Aβ. After IP, Aβ bound gangliosides were detected via TLC. GM3 binds to synthetic Aβ40 and Aβ42 (shown for equimolar concentrations), and cellular derived Aβ40 and Aβ42 (shown for physiological concentrations, approx. 10∶1 ratio). Thin black vertical lines (Fig. 2D left) indicate that the TLC plates were scraped to separate lines. (*E*) Co-IP of Aβ and GD3S in dependence of GM3. Aβ only binds to GD3S in presence of the substrate GM3. Data are represented as mean +/− SEM.

To identify the molecular mechanism of APP mediated GD3S regulation, we analyzed the potential role of the APP cleavage products Aβ and AICD, which are missing in PS- or APP-deficient cells. To identify whether Aβ peptides may influence GD3S activity, MEF PS1/2-/- and MEF APP/APLP2-/- cells were incubated with synthetic Aβ40 or Aβ42 peptides at physiological peptide concentrations. Silver stain analysis of the Aβ peptides revealed soluble monomeric Aβ, but no stable small oligomers (Fig. S3). Incubation with synthetic soluble Aβ40 or Aβ42 peptides resulted in decreased GD3S activity, both in cells lacking either PS1/2 or APP/APLP2 (Fig. 2*A*). To investigate whether the effect of Aβ on GD3S is due to a direct interaction, homogenates of MEF PS1/2-/- cells and a cell-free assay, which only contained purified GD3S and the GD3S substrate GM3, were incubated with Aβ and GD3S activity was determined. Both cell homogenates and the cell-free assay showed significantly decreased GD3S activity in presence of Aβ (Fig. 2*B*), suggesting that decreased GD3S activity is mediated directly by the interaction of Aβ with components of the cell-free assay. Inverted Aβ peptides and aggregated Aβ had no influence on GD3S activity (Fig. 2*C*), indicating a specific requirement for soluble Aβ peptides as expected for a physiological regulatory mechanism. Since Aβ peptides were able to decrease GD3S activity in a cell-free assay, consisting only of the substrate GM3 and the enzyme GD3S, two molecular mechanisms explaining how Aβ influences GD3S activity are conceivable: Firstly, Aβ could bind to the substrate, thus reducing substrate availability of the enzyme or secondly in principle, Aβ could directly bind to the enzyme, reducing the turnover of GM3 to GD3. Of interest in the first context is that Aβ is known to bind to GM1, another *a*-series ganglioside [24]. Indeed, Aβ co-immunoprecipitated GM3, the substrate for GD3S, but interestingly, it did not bind GD3, the product of GD3S (Fig. 2*D*, Fig. S4A, S4 *B*). In agreement with our previous results we found, using synthetic Aβ40 and Aβ42 peptides and the Aβ40- and Aβ42-specific antibodies G2-10 and G2-11 for co-immunoprecipitation (co-IP), that both Aβ species bind GM3 (Fig. 2*D*; Fig. S4 *C*). To rule out effects caused by the use of synthetic rather than naturally derived Aβ peptides, we analyzed conditioned media of APP expressing cells, secreting Aβ40 and Aβ42 in a ratio of approximately 10∶1 [30]. The experiments with conditioned media for co-IP confirmed that naturally derived Aβ40 and Aβ42 bind GM3 at a similar 10∶1 ratio (Fig. 2*D*; Fig. S4 *B*). As a positive control we used GM1, which is known to bind Aβ [23], [24], [25]. GM1 bound to both Aβ species, Aβ40 and Aβ42 (Fig. S4 *D*). These experiments suggest that these GM3-Aβ complexes are mechanistically involved in the reduced GD3S activity we observed in the APP or PS knock-out experiments. As already mentioned, a second mechanism could be that Aβ directly binds to GD3S and in this way reduces the turnover of GM3 to GD3. Co-IP studies revealed that Aβ was not able to bind to GD3S in the absence of GM3, ruling out that Aβ reduces GD3 production by direct binding to the enzyme (Fig. 2*E*). Importantly, under the same experimental conditions, but in presence of GM3, Aβ was bound to GD3S (Fig. 2*E*). This emphasizes that the inhibition of GD3S is mediated by binding of Aβ to GM3. When the GM3-Aβ complex binds to GD3S, as indicated by Fig. 2*E*, the conversion of GM3 to GD3 is reduced or prevented, consequently reducing substrate availability. Additionally, the GM3-Aβ complex might compete with unbound GM3 for GD3S binding and therefore acts as a competitive inhibitor, which would further reduce the amount of GM3 available for GD3S.

### AICD decreases GD3-synthase gene expression

**Figure 3:**
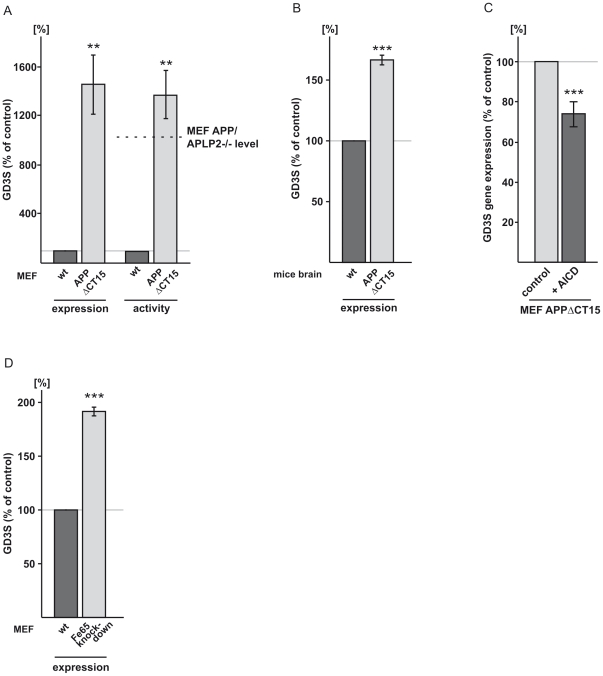
Effect of the APP intracellular domain (AICD) on GD3S. (*A*) MEF, deficient in full-length APP expressing a truncated APP construct lacking 15 aa from the C-terminus (APPΔCT15) show increased GD3S expression and activity. The level of the GD3S activity of the APP/APLP2 knock-out cells (MEF APP/APLP2-/-) are indicated (horizontal dotted black line). Interestingly the MEF APP/APLP2 knock-out level showed a slight less effect strength compared to the MEF APPΔCT15 cells. However this difference did not reach a significant level and might be due to clonal heterogeneity. (*B*) Increased GD3S expression in APPΔCT15 mouse brains. (*C*) GD3S gene expression: AICD peptide partially rescues elevated GD3S gene expression in MEF cells expressing the C-terminal truncated APP. An AICD peptide consisting of the last 20 aa from the APP C-terminus (AICD) is able to decrease GD3S expression. (*D*) shRNA generated Fe65 knock-down cells show increased GD3S gene transcription.

In addition to the Aβ-mediated reduction in GD3S activity, a further mechanism of action of influencing GD3S gene transcription, apparently exists. This is evidenced by the observation that PS- and APP/APLP2-deficient cells as well as brains of APP-/- mice showed elevated GD3S gene expression (Fig. 1*C*, *D*, *E*). In accordance with this, Aβ only partially rescued the increased GD3S activity in PS- or APP-deficient MEF (Fig. 2*A*), indicating that besides Aβ other PS- and APP-dependent factors are involved in GD3S activity regulation. The intracellular domain of APP (AICD) is assumed to contribute to the regulation of gene expression [31], [32] comparable to the function of the Notch intracellular domain (NICD), which is also released by γ-secretase [33]. To investigate the effect of AICD on GD3S gene expression we analyzed APP knock-in mouse embryonic fibroblasts deficient of full-length APP, expressing an APP construct, that lacks the last 15 aa from the C-terminus (MEF APPΔCT15) and hence a functional AICD domain [34]. The deleted last 15 aa include the YENPTY motif of APP, which apparently plays a crucial role in regulating gene transcription by binding to Fe65/X11 [31]. Indeed, MEF APPΔCT15 cells showed increased GD3S gene transcription and a nearly identical increase in GD3S activity (Fig. 3*A*). Supporting the *in vivo* relevance of these findings, brains of mice expressing APPΔCT15 also showed increased GD3S expression (Fig. 3*B*), which is in line with the result of APP knock-out mice (Fig. 1*E*). These results indicate that the presence of AICD strongly modulates GD3S gene transcription. To exclude that altered Aβ production, which might be caused by the truncated APP construct APPΔCT15 [35], [36], could be responsible for the observed effect, we incubated MEF APPΔCT15 cells with a synthetic AICD peptide, corresponding to the last 20 aa of the C-terminus of APP, which was applied together with SAINT-2:DOPE for efficient peptide uptake [37]. Indeed, the synthetic peptide significantly decreased GD3S expression in MEF APPΔCT15 cells (Fig. 3*C*), validating a function of AICD in regulating GD3S gene transcription. To further verify the role of AICD in regulating gene expression of GD3S, we generated Fe65 knock-down cells. As expected, a 58% reduction of Fe65 expression (Fig. S5), was sufficient to almost double GD3S expression levels (Fig. 3*D*).

As described above, Aβ directly causes a decrease in GD3S activity. To evaluate if, beside the observed AICD effect on gene expression, AICD has a similar direct effect on GD3S activity, homogenates of MEF APPΔCT15 cells were incubated with AICD peptides. Because homogenated cells were used for this experiment, alterations in GD3S activity cannot be caused by an AICD-induced change in GD3S gene expression. No effect of AICD peptide on GD3S activity was observed in these cell homogenates, indicating that AICD has no influence on GD3S enzymatic activity directly (Fig. S6 *A*). Additionally, an influence of Aβ peptides on GD3S gene expression was ruled out by analyzing GD3S expression in PS-deficient cells supplemented with Aβ40 or Aβ42 (Fig. S6 *B*).

**Figure 4:**
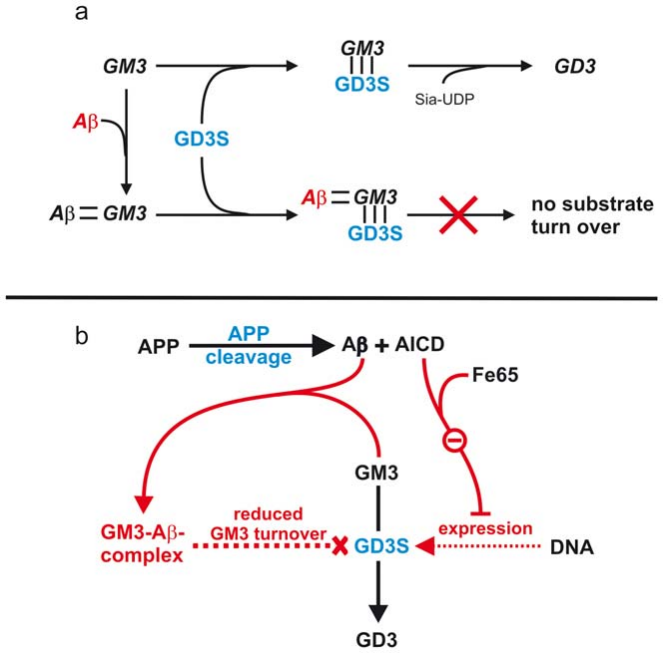
Molecular mechanisms of APP cleavage products in the regulation of GD3S enzyme activity. (*A*) In absence of Aβ peptides *a*-series ganglioside GM3 binds to GD3S and is converted to the *b*-series ganglioside GD3. In presence of Aβ, Aβ binds ganglioside GM3, forming an Aβ-GM3 complex. This complex still binds to GD3S, but cannot be converted to GD3. (*B*) Dual function of Aβ and AICD in GD3S regulation. Aβ reduces enzyme activity of GD3S by forming an Aβ-GM3 complex, resulting in reduced turnover of GM3 to GD3. AICD binds the adaptor protein Fe65 and reduces GD3S gene transcription, which also results in reduced turnover of GM3 to GD3.

Taken together, a dual mechanism of GD3S regulation mediated by PS-dependent APP processing can be postulated. Aβ peptides bind to GM3, thus decreasing GD3S activity by reducing substrate availability (Fig. 4*A*), whereas AICD inhibits gene expression of GD3S (Fig. 4*B*). Both mechanisms synergistically result in decreased cellular GD3S activity and thus in increased GM3 and reduced GD3 levels and an altered ratio of *b*∶*a* series gangliosides.

### GM3 decreases, GD3 increases Aβ production

**Figure 5:**
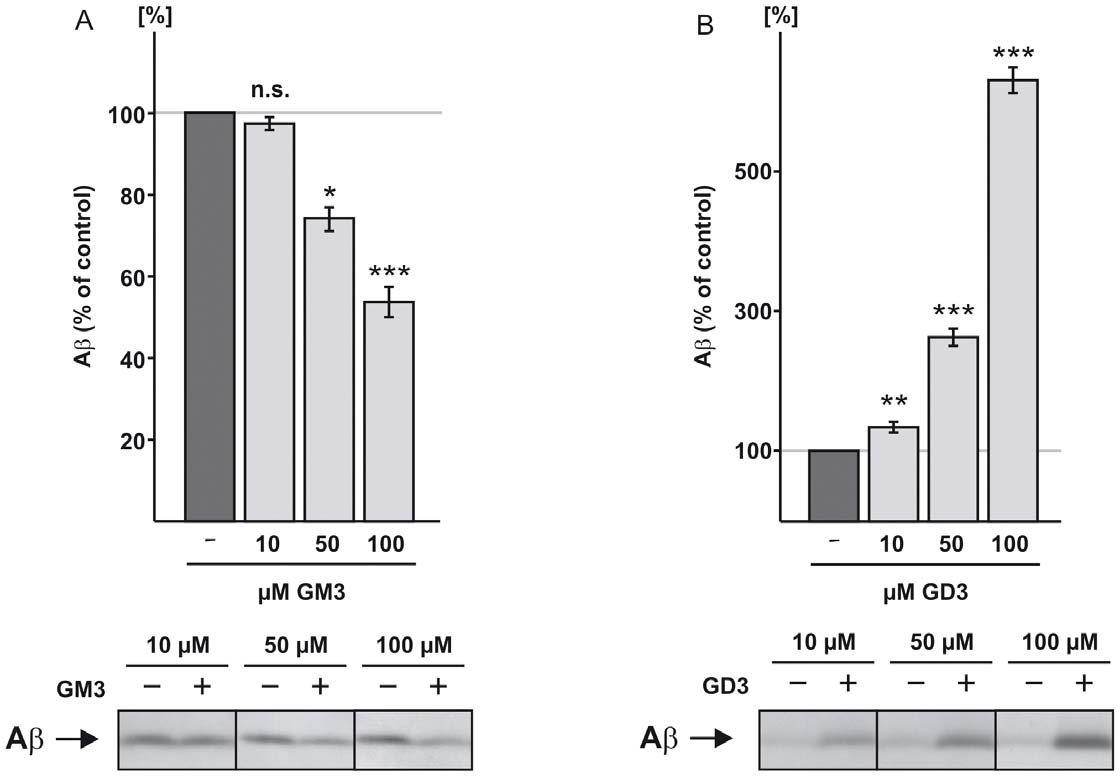
Effect of GM3 and GD3 on APP processing. (*A*) Dose-dependent effect of GM3 on Aβ production: COS7 cells stably transfected with SPC99, representing the β-secretase cleaved C-terminal fragment of APP, show a dose-dependent decrease in Aβ production in presence of GM3. Aβ levels were determined by IP and Western blot (WB) analysis. (*B*) Dose-dependent effect of GD3 on Aβ generation in SPC99 expressing COS7 cells. GD3 enhances Aβ generation dose-dependently. Aβ levels were determined by IP and WB analysis.

It has been shown that the ganglioside GM1 increases Aβ production [22]. In order to analyze the effect of GM3 and GD3, which are themselves affected by APP processing, on Aβ generation and to evaluate the presence of a potential regulatory cycle in ganglioside homeostasis and APP processing, we incubated COS7 cells, stably transfected with the truncated APP construct SPC99, with GM3 or GD3. This shortened APP construct represents the C-terminal fragment of β-secretase cleaved APP, and allows the study of GM3 and GD3 influence on γ-secretase activity, independent of β-secretase activity [38], [39]. Unexpectedly, the ganglioside GM3 decreased (Fig. 5*A*), but GD3 increased Aβ levels (Fig. 5*B*). Both effects were dose-dependent in a range from 10 to 100 µM. LDH-assay analysis revealed no signs of elevated cytotoxicity or reduced membrane integrity in the presence of gangliosides (Fig. S7). The effect of GM3 and GD3 is apparently cell line independent, since we obtained similar results in the human neuroblastoma cell line SH-SY5Y (Fig. S8).

## Discussion

Gangliosides are sialic acid containing glycosphingolipids ubiquitously present in eukaryotic membranes with numerous cellular functions like signal transduction, cell adhesion, protein transport and brain development [17]. The numerous functions of gangliosides and the sequential anabolic pathway as described in Fig. 1*A* suggest that these enzymes must be tightly regulated. The major brain gangliosides belong either to the *a*- or *b*-series of gangliosides, emphasizing the importance of GD3S for brain ganglioside homeostasis. Although it is well established that brain ganglioside composition alters continuously during aging and in AD [19], [28], the underlying cellular mechanisms remain unknown. To evaluate the link between ganglioside metabolism, APP processing and the resulting consequence for AD pathology, we analyzed ganglioside homeostasis for dependence on APP processing.

Absence of either APP or PS in cells or in the corresponding mouse models increased GD3S activity and expression, which, as a consequence, resulted in an increase in the *b*- to *a*-series ratio of gangliosides. Breaking down the molecular mechanism in detail, we identified that the regulation of GD3S by APP processing depends on two molecular factors, Aβ peptides and AICD. Aβ selectively binds GM3, the GD3S substrate, but not GD3, the GD3S product. The GM3-Aβ complex is still able to bind to GD3S but Aβ effectively prevents the conversion of GM3 to GD3, hence resulting in lowered cellular GD3S activity. Because Aβ is secreted and taken up by cells [40], [41] and since there are, especially in neural cells, abundant amounts of Aβ present intracellularly [42], [43], [44], binding of Aβ to GM3 could in principle take place in any intracellular compartment, especially along the secretory pathway and endosomes, at the plasma membrane, where large amounts of Aβ are present, as well as in the extracellular space [45]. Selective reduction of substrate availability by binding of the substrate to an inhibitor, often described as substrate depletion, is a common molecular mechanism to modulate substrate turnover. A similar mechanism is described for phospholipids, which can be cleaved by phospholipases (PLA2). In the presence of the protein annexin, PLA2 activity is reduced by binding of annexin to the phospholipids [46]. Interestingly, Aβ has been observed to bind several other lipids, such as cholesterol [47] and GM1 [24]. GM1, another *a*-series ganglioside, is elevated in amyloid plaques further strengthening the link between GD3S regulation and AD [48]. With this conditions used here neither synthetic nor naturally derived soluble Aβ bound GD3. It has previously been reported that synthetic Aβ 1–40 as well as synthetic inverted Aβ 40-1 binds to all sialylated gangliosides [49]. This might indicate that conditions exist, such as amyloid formation as typical for AD, under which Aβ like peptides have increased affinity to all sialylated gangliosides. In such a case, binding of GD3 to Aβ, would likely further contribute to the disturbance of the physiological ganglioside homeostasis in the AD brain.

Reduced GM3 to GD3 turnover is further enhanced by another Aβ independent effect. Although Aβ directly decreased GD3S activity, this cannot account for the observed increase in GD3S expression. Addition of Aβ to the knock-out cells had no influence on GD3S expression levels. Cao and others have found that AICD has structural and functional similarities with NICD, a well-established regulator of gene transcription [50]. However, the relevance of AICD for transcriptional regulation remains controversial [12], [31], [32], [50]. To elucidate whether the increased GD3S expression is mediated *via* AICD, we used APPΔCT15 cells lacking the last 15 aa of the APP C-terminus and therefore a functional AICD. MEF APPΔCT15 cells showed drastically elevated GD3S gene transcription and similarly increased GD3S activity. Confirming the essential role of a functional AICD for GD3S regulation, the increased GD3S expression in APPΔCT15 cells could be partially rescued by addition of synthetic AICD peptide. To further verify our results that AICD triggers a cascade that results in decreased GD3S expression, we investigated whether a similar effect can be obtained by eliminating another protein from this cascade. Fe65 binds to the YENPTY motif of AICD and mediates nuclear targeting of AICD [51]. In line with the findings that absence of AICD causes an increase in GD3S expression, we found in Fe65 knock-down cells increased GD3S expression, validating the AICD/Fe65-mediated mechanism for regulating GD3S expression. Similar to the finding that Aβ altered GD3S enzymatic activity, but not GD3S expression, AICD suppressed GD3S expression, but had no influence on GD3S enzymatic activity.

These findings support an essential role of APP in ganglioside homeostasis in AD. The function of AICD, Aβ and γ-secretase in regulating GD3S would suggest that the alteration in ganglioside composition is a consequence of AD. Nevertheless, both the substrate and product of the GD3S, GM3 and GD3, themselves modulate APP processing making the distinction between cause and consequence less clear. Like ganglioside GM1, GD3 increased Aβ generation whereas GM3 decreased Aβ release. Glycosphingolipids have been shown to be implicated in the regulation of the subcellular transport of APP in the secretory pathway [52], indicating that altered Aβ generation in presence of different gangliosides might be caused by altered APP transport to the cellular compartments where Aβ generation preferentially occurs, in post-Golgi secretory and endocytic compartments [45], [53]. In addition changes in lipid raft composition might alter Aβ generation. For example it has been recently shown that docosahexaenoic acid (DHA) decreases Aβ generation by decreasing cholesterol in lipid raft membrane microdomains [54]. The hypothesis that lipids are also important for functional cellular protein transport as already reported for glycosphingolipids by Tamboli et al. [52] is further substantiated by the finding that BACE1 protein transport to the endosomal compartments, where β-secretase cleavage preferentially occurs, is impaired in presence of some lipids, e.g. DHA [54]. However, although it has been shown that membrane lipid composition influences APP cleavage [7], [8], [9], [10], [11], we do not exclude that lipids, such as GM3 and GD3, might directly affect secretase activities or other cellular mechanisms involved in Aβ generation. Reducing GD3S activity as potential therapeuthic target to treat or prevent AD would result in decreased GD3 and increased GM3 levels both resulting in decreased Aβ levels. However, one has to take into consideration that GM3 is further converted to ganglioside GM1, which is discussed to increase Aβ generation and to induce Aβ aggregation *in vitro*. In opposite to the *in vitro* findings concerning GM1 induced Aβ aggregation, *in vivo* studies revealed that GM1 is not required for Aβ aggregation [55] or even neuroprotective [14], [56], [57]. Disruption of the GM2 synthase gene in APP transgenic mice significantly increased Aβ aggregation, although GM1 is completely lacking in these mice [55]. Moreover, Bernardo et al. observed that deletion of GD3S, increasing neuronal expression of GM1, results in reduced soluble Aβ levels, decreased Aβ aggregation and plaque load in APP/PS transgenic mice paralleled by behavioural improvements [14]. From this line of evidence changes in the cerebral ganglioside composition might alter Aβ generation and aggregation and might be beneficial regarding AD pathology. Additionally, a recent study from Dhanushkodi et al. reports neuroprotective effects *in vivo* using sialidase from Vibrio cholerae producing a brain ganglioside profile similar to that of the GD3S knock-out, further implicating GD3S as a potential therapeutic target for AD [58]. However, further experiments are necessary to identify the role of specific gangliosides in AD pathology.

**Figure 6:**
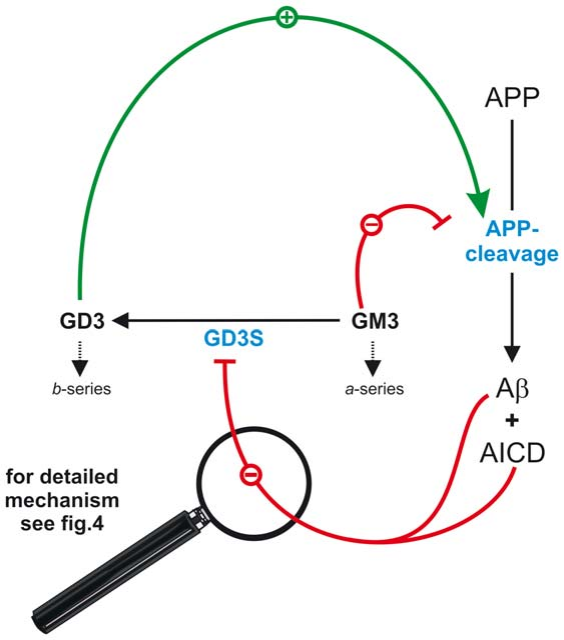
Hypothetic model of the physiological functions of Aβ and AICD in the regulation of GD3-synthase (GD3S) – the enzyme controlling major brain ganglioside composition. (*A*) Amyloidogenic proteolytic processing of the Alzheimer's amyloid precursor protein (APP) releases amyloid-beta peptides (Aβ) and the intracellular domain of APP (AICD). Aβ and AICD inhibit GD3S, resulting in reduced conversion of *a*- to *b*-series gangliosides. As a consequence of reduced conversion of *a*- to *b*-series gangliosides, GM3 increases whereas GD3 decreases. In return, both gangliosides, GM3 and GD3, themselves regulate the proteolytic cleavage of APP. The *b*-series ganglioside GD3 increases, whereas the *a*-series gangliosides GM3 decreases amyloidogenic proteolytic processing of APP.

From our findings we propose a hypothetic model showing that GD3S and APP form a regulatory feedback cycle that links ganglioside metabolism with APP processing (Fig. 6). Since this regulatory cycle involves synergistic components the magnitude of the effect can be increased. Both Aβ and AICD decrease GD3S activity, resulting in strongly decreased GD3 levels. This in turn leads to reduced cleavage of APP. Additionally, decreased GD3S activity results in increased GM3 levels, further reducing APP cleavage to Aβ. It can therefore be proposed that interventions shifting the GD3/GM3 ratio towards increased GM3 and derceased GD3 levels could, by influencing this regulatory feedback cycle, cause reduced amyloidogenic processing of APP. This therefore may prove to be a potential strategy to treat or prevent AD.

## Materials and Methods

### Cell culture and biological material

COS7, SH-SY5Y and MEF cells were cultivated in DMEM (Sigma, Taufkirchen, Germany), 10% FCS (PAN Biotech, Aidenbach, Germany); for pCEP4/APP and pCEP4/SPC99 transfected cells additional HygromycinB (400 µg/ml) (PAN Biotech, Aidenbach, Germany) [44] was used. MEF PS1/2-/- cells were transfected with pCNA3.1/PS1 wildtype using Superfect as described by the manufacturer (Qiagen) to generate PS1 wildtype retransfected control cells (MEF PS1r). Stable transfectants were selected using 300 µg/ml Zeocin. MEF PS1r cells were cultivated in DMEM (Sigma, Taufkirchen, Germany), 10% FCS (PAN Biotech, Aidenbach, Germany), 300 µg/ml Zeocin (Invitrogen, Karlsruhe, Germany). Stable transfection and functionality of MEF PS1r cells were validated by PS1 western blot analysis (Fig. S1A), Aβ generation (Fig. S1B) and enzymatic measurement of γ-secretase activity (Fig. S1C). MEF PS1/2-/- cells [26], [27], MEF PS1r cells [10], MEF APP/APLP2-/- cells [59] and MEF APPΔCT15 cells [34] have been described previously.

Ganglioside (Axxora, Lörrach, Germany) exposure was carried out in culture media without FCS for 16+6 hours. Notably the critical micelle concentration (CMC) of gangliosides examined in previous studies varies in dependence of the used methods. Whereas one study reports that for all gangliosides the CMC is between 70 to 100 µM [60], another study suggests for monosialogangliosides a CMC of 8.5*10^−5^ M and for disialogangliosides a CMC of 9.5*10^−5^ M [61]. In addition a more recent study suggests an even lower CMC for ganglioside GM3 of 3.4*10^−9^ M [62]. Therefore we used different ganglioside concentrations from 10 to 100 µM, which revealed a dose-dependent effect (Fig. 5).

### Western Blot analysis

Aβ analysis of conditioned media of cultured cells was performed with antibody W02 as described earlier [63]. Control experiments using antibody WO2 for Aβ detection are shown in supplement Figures S9A, S9B and S9C. For the detection of PS1, cell-lysates were separated on 10–20% Tricine gels (Anamed, Groß-Bieberau, Germany). Cell-lysates were generated as previously described [44]. WB analysis was performed with the antibody sc-7860 (1∶500; Santa Cruz, Heidelberg, Germany).

Densiometric quantification was performed using Image Gauge software. Samples were adjusted to equal protein amount before WB analysis [64].

### Ganglioside determination and thin layer chromatography

After washing confluent grown cells three times with ice-cold phosphate-buffered saline (PBS), cells were scraped off and homogenized using a PotterS (B. Braun, Melsungen, Germany) at maximum speed (30 strokes). Protein amount was adjusted to 70 mg/ml according to Smith *et al.* [64]. After protein adjustments, a modified Bligh & Dyer method [65] was used to isolate gangliosides. After desalting the lipids via a reversed-phase cartridge (Waters Oasis, Eschborn, Germany) according to Whitfield *et al.* [66] a highly concentrated extract was applied to silica gel thin-layer chromatography (TLC) plates (Merck, Darmstadt, Germany). As a solvent system CHCl_3_/MeOH/H_2_O-CaCl_2_ 0.2% (60/35/8) was used [67]. The glycolipids were visualized by iodine and identified by their Rf-values and commercially available standards (AvantiPolarLipids, Alabaster, USA). Densiometric quantification was performed using Image Gauge software. All solvents used were HPLC grade (VWR International GmbH, Darmstadt, Germany). To validate the use of iodine to stain gangliosides, we separated brain samples and ganglioside standard via TLC and stained either with iodine, orcinol or resorcinol (Fig. S10).

### Determination of binding properties of Aβ to gangliosides and GD3S

Co-IP of Aβ with gangliosides was carried out with Aβ, produced by SH-SY5Y cells stably transfected with APP695 or with synthetic Aβ peptides (10 ng/ml) (B. Penke, Szeged, Hungary). For immunoprecipitation of Aβ peptides bound to gangliosides, monoclonal antibodies W02 (1 µg/ml), G2-10 (12.5 µg/ml), specific for the detection of Aβ40, and G2-11 (17.3 µg/ml), specific for the detection of Aβ42, were used [63]. Gangliosides bound to Aβ peptides were analyzed by TLC. Purified GD3S was purchased from Abnova (Taipei, Taiwan), used at a final concentration of 0.5 µg/ml and detected by silverstain [68].

### Co-Immunoprecipitation of GD3S with Aβ peptide and Ganglioside GM3

13.65 nM of GD3S (Abnova, Taipei, Taiwan), 11.55 nM Aβ40, 1.155 nM Aβ42 were preincubated in PBS (pH 7.0, final volume 1 ml) in absence or in presence of 100 µM GM3 in a glass tube for 3 hours at 4°C with gentle shaking. After preincubation, samples were transferred in a microcentrifuge tube and 5 µg of antibody WO2 and 20 µl ProteinG-Sepharose were added. Immunoprecipitation of Enzyme-Peptide-Lipid-complex was performed over night at 4°C on an end over end shaker. At the end of incubation time, samples were centrifuged at 13.000 rpm for 1 min at 4°C. Supernatant was removed and Sepharose Beads were washed three times with 1 ml of 10 mM Tris (pH 7.4) Samples were boiled in SDS-sample buffer (187.5 mM Tris/HCl pH 6.8, 6% SDS, 30% Glycerol, 15% β-Mercaptoethanol 0.03% Bromphenolblau) and separated on a 10–20% Tris-Tricine Gel. Proteins were detected by silver staining according to the method of Switzer et al. [68].

### Determination of monomeric Aβ40 and Aβ42 by SDS PAGE and Silver Staining

To determine the monomeric status of Aβ40 and Aβ42, used in *in vitro* and cell culture experiments, the synthetic peptides (200 ng Aβ40+20 ng Aβ42) were separated by SDS PAGE on a 12% NuPAGE Bis-Tris gel (Invitrogen), according to Dahlgren et al., which were also able to detect Aβ oligomers by utilizing this method [69]. Proteins were visualized by silver stain.

### Enzymatic assays

For measuring **GD3S** activity confluent grown cells were washed three times with ice-cold sodium cacodylate 7.5 µM pH 5.8 including 1.5% Triton X-100, scraped off and homogenized using a PotterS (B. Braun, Melsungen, Germany) at maximum speed (100 strokes) [70]. After protein adjustment to 25 mg the reaction was started by addition of 5 nM CMP-sialic acid [^3^H] (ARC, St. Louis, USA), 10 nM GM3 (Axxora, Lörrach, Germany) and 50 nM CMP-sialic acid (Sigma, Taufkirchen, Germany) in sodium cacodylate 7.5 µM pH 5.8 at 37°C. After 45 min the enzymatic reaction was stopped by addition of MeOH and CHCl_3_, gangliosides were extracted as described above and separated via TLC. The GD3-band was scraped off and the including radioactivity was measured via scintillation counting in Tri-Carb2800TR (Perkin Elmer, Rodgau-Jügesheim, Germany). To determine the effect of Aβ40 (10 ng/ml), Aβ42 (1 ng/ml) (B. Penke, Szeged, Hungary), inverted Aβ (10 ng/ml) or AICD (2 µM) (Genscript Corporation, Piscatway, USA) synthetic peptides were incubated for 1 h to cell-lysate and for 9 days in cell culture. After incubation the reaction was started by adding 5 nM CMP-sialic acid [^3^H] (ARC, St. Louis, USA), 10 nM GM3 (Axxora, Lörrach, Germany) and 50 nM CMP-sialic acid (Sigma, Taufkirchen, Germany) in sodium cacodylate 7.5 µM pH 5.8 to the preincubated GM3-Aβ complex and stopped after another 45 min by addition of MeOH.

For the **GD3S** ***in vitro*** **assay** 25 nM Aβ and 5 nM GM3 were preincubated for 1 h to allow the formation of the complex GM3-Aβ. After that the reaction was started by adding 5 nM CMP-sialic acid [^3^H] (ARC, St. Louis, USA), 10 nM GM3 (Axxora, Lörrach, Germany) and 50 nM CMP-sialic acid (Sigma, Taufkirchen, Germany) in sodium cacodylate 7.5 µM pH 5.8 to the preincubated GM3-Aβ complex and stopped after another 45 min by addition of MeOH. For cell-free enzymatic assay purified GD3S (1 µg/ml; 27.3 nM) (Abnova, Taipei City, Taiwan) was used instead of lysate. Aggregated Aβ was prepared as 22.2 µM solution by incubation >24 h at 37°C [10] and finally used as 22.2 nM. All solvents used were HPLC grade (VWR International GmbH, Darmstadt, Germany). GD3S assay unspecificity was determined using ganglioside GM2 (Fig. S11).

### Determination of γ-secretase activity

Detection of γ-activity was performed as described before [38], [54]. Briefly, cells were washed three times with ice-cold phosphate-buffered saline (PBS), scraped off in sucrose buffer (10 mM Tris/HCl pH 7.4 including 1 mM EDTA and 200 mM sucrose) and homogenized using a PotterS at maximum speed (25 strokes). After protein adjustment to 1 mg according to Smith *et al.* 1985 [64], the samples were centrifuged at 900rcf for 10 min at 4°C and the obtained post-nuclear fractions were ultracentrifuged at 55000 rpm for 75 min at 4°C. Pelleted membranes were resuspended using cannulaes with decreasing diameter in 300 µl sucrose buffer. The volume is partioned in 96well plates (γ: 100 µl, equates to 250 µg protein) and γ-secretase substrate [71] (Calbiochem, Darmstadt, Germany) (10 µM) was added. Fluorescence was measured for 3 h under light exclusion using excitation at 355±10 nm and fluorescence detection at 440±10 nm or 345±5 nm/500±2.5 nm respectively. Assay specificity is >90% for γ-secretase activity assay, and was validated using γ-secretase inhibitor L-658458 (Merck, Darmstadt, Germany).

### Knock-down experiments

According to the manufacturer's protocol SureSilencing™ shRNA Plasmid (SABioscience, Frederick, USA) was used. The following insert sequences were used:

Fe65: 5′-TCC CTG GAC CAC TCT AAA CTT-3′

5′-CAA CCC AGG GAT CAA GTG TTT-3′

5′-AAG GCT TTG AGG ATG GAG AAT-3′

5′-TGT CCA CAC GTT TGC ATT CAT-3′.

Control: 5′-GGA ATC TCA TTC GAT GCA TAC-3′.

This control was used as randomized sequence in all knock-down experiments. The knock-down was verified by quantitative real-time experiments.

### Quantitative real-time experiments

Total RNA was extracted from cells or tissue using RNeasyPlus Mini Kit (Qiagen, Hilden, Germany) or TRIzol reagent (Invitrogen, Karlsruhe, Germany), using manufacturer's protocols. 2 µg were reverse-transcribed using High Capacity cDNA Reverse Transcription Kits and quantitative real-time PCR analysis was carried out using Fast SYBR Green Master Mix on 7500 Fast Real-Time PCR System (7500 Fast System SDS Software 1.3.1.; Applied Biosystems, Darmstadt, Germany). As control RNA samples were normalized to β-actin gene expression.

To determine the effect of Aβ40 (10 ng/ml), Aβ42 (1 ng/ml) (B. Penke, Szeged, Hungary) or AICD on gene transcription (2 µM) (Genscript Corporation, Piscatway, USA) synthetic peptides were incubated for 9 days in cell culture. Alternatively, the compound SAINT-2:DOPE was added together with the synthetic peptides for 12 h to the cells to achieve efficient delivery of the peptides [37].

Changes in gene expression was calculated using 2^−(ΔΔCt)^ method [72]. The ΔΔCT data are shown in table S1. To verify the results obtained by quantitative real-time experiments, samples were separated on 1.5% agarose gels in TBE buffer (90 mM Tris, 90 mM boric acid, 2 mM EDTA pH 8.0).

The following primer sequences were used:

Fe65 pair1: 5′-TCT TGC ACC AGC AGA CAG AG-3′ and

5′-CAG CCA TGA TGA ATG CAA AC-3′;

pair2: 5′- TTT GGA AGG ATG AAC CCA GT-3′ and

5′-AAG CTT CTC CTC CTC TTG GG-3′;

pair3: 5′- GCT CTA AGA TCA TGG CCG AA-3′ and

5′-GGA ATT CCA CTT GGA AAG GG-3′.

β-Actin: 5′-CCT AGG CAC CAG GGT GTG AT-3′ and

5′-TCT CCA TGT CGT CCC AGT TG-3′.

### Cytotoxicity measurement

Cytotoxicity was measured using Lactate Dehydrogenase Cytotoxicity Assay Kit (LDH-Assay) and performed as described in the manufacturer's protocol (Cayman Chemical, Ann Arbor, USA). Briefly, conditioned media of incubated cells were centrifuged for 5 min at 400 g. 100 µl supernatant was used to determine lactate dehydrogenase cytotoxicity levels. For each cytotoxicity measurement lactate dehydrogenase standards were used as described in the manufacturer's protocol. After adding 100 µl reaction solution, the plate was agitated for 30 min at RT. Absorbance was measured at 490 nm using a MultiskanEX plate reader (Thermo Fisher Scientific, Waltham, USA).

### Statistical analysis

All quantified data represent an average of at least three independent experiments. Error bars represent standard deviation of the mean. Statistical significance was determined by two-tailed Student's t-test; significance was set at *P≤0.05; **P≤0.01 and ***P≤0.001, n.d. = not detectable.

## Supporting Information

Figure S1 Analysis of PS1 retransfected mouse embryonic fibroblasts devoid of PS1 and PS2. (*A*) PS expression in mouse embryonic fibroblasts (MEF) devoid of PS1 and PS2 (PS1/2-/-), corresponding wild-type embryonic fibroblasts (wt) and MEF PS1/2-/- cells retransfected with PS1 (PS1r). Western blot analysis of cell-lysates; PS was detected using the antibody sc-7860. (*B*) Determination of Aβ generation in PS1 retransfected MEF PS1/2-/-. MEF PS1/2-/- and MEF PS1r were infected with Semliki Forest Virus expressing APP695. Conditioned media were collected, immunoprecipitated with antibody W02 and detected via WB analysis using W02. (*C*) γ-secretase activity of MEF wild-type (wt) cells and MEF PS1r. γ-secretase activity of MEF wt and MEF PS1r was measured as described in Material and Methods.
(TIF)

Figure S2 TLC analysis of *a*- and *b*-series gangliosides in PS-deficient cells. (A) A representative TLC, analyzing the ganglioside pattern in PS1/2 deficient mouse embryonic fibroblasts (MEF PS1/2-/-) and MEF PS1/2-/- retranfected with PS1 wildtype (MEF PS1r) is shown. A ganglioside standard was loaded on the TLC. (B) Densiometric quantification of a representative TLC.
(TIF)

Figure S3 Determination of monomeric Aβ40 and Aβ42 by SDS Page and Silver staining. Synthetic Aβ40 and Aβ42 peptides (200 ng Aβ40, 20 ng Aβ42) used for the *in vitro* and cell culture experiments were loaded and separated on a 12% NuPAGE Bis-Tris gel Proteins were visualized by silver stain.
(TIF)

Figure S4 Co-immunoprecipitation of gangliosides with Aβ40 and Aβ42 peptides. (A) Co-immunoprecipitation studies using ganglioside GD3, cellular derived Aβ40 and Aβ42 peptides and the Aβ40 and Aβ42 specific antibodies G2-10 and G2-11. Cellular derived Aβ40 and Aβ42 peptides were obtained from conditioned media of APP wildtype expressing cells. Ganglioside GD3 does not co-immunoprecipitates with Aβ40 and Aβ42. (B) Co-immunoprecipitation studies using ganglioside GM3, cellular derived Aβ40 and Aβ42 peptides and the Aβ40 and Aβ42 specific antibodies G2-10 and G2-11. Cellular derived Aβ40 and Aβ42 peptides were obtained from conditioned media of APP wildtype expressing cells, secreting Aβ40/Aβ42 peptides in a ratio of approximately 10∶1. Ganglioside GM3 co-immunoprecipitates with Aβ40 and Aβ42. In accordance to the 10∶1 ratio of cellular derived Aβ40/Aβ42 peptides, the detected GM3 band shows a similar 10∶1 ratio. (C) Co-immunoprecipitation studies using ganglioside GM3 and equimolar concentrations of synthetic Aβ40 and Aβ42 peptides. GM3 co-immunoprecipitates with synthetic Aβ40 and Aβ42 peptides. As expected using equimolar concentrations of Aβ40 and Aβ42 peptides, the stained (co-immunoprecipitated) GM3 bands have nearly identical intensity. (D) Co-immunoprecipitation studies using ganglioside GM1 and equimolar concentrations of synthetic Aβ40 and Aβ42 peptides. Black dotted vertical line: Samples were loaded on the same TLC but were not next to each other.
(TIF)

Figure S5 Fe65 knock-down in human neuroblastoma SH-SY5Y cells. The Fe65 expression level measured by RT-PCR analysis was reduced to 42%. Corresponding agarose gel is shown.
(TIF)

Figure S6 Influence of AICD on GD3S activity and of Aβ peptides on GD3S expression. (*A*) AICD peptides were incubated on APPΔCT15 cell homogenates and GD3S activity was determined as described. (*B*) Aβ peptides (Aβ40 and Aβ42) were incubated in cell culture on MEF PS1/2-/-. GD3S expression was determined by RT-PCR.
(TIF)

Figure S7 Lactate dehydrogenase assay in cells incubated with 100 µM GM3 or 100 µM GD3 versus corresponding solvent control (ddH_2_O). No signs for increased cytotoxicity were observed in cells treated with gangliosides.
(TIF)

Figure S8 Aβ production in SPC99 expressing SH-SY5Y cells incubated with GM3 and GD3. Cells were incubated with 100 µM GM3 and 100 µM GD3, respectively, versus corresponding solvent control (ddH_2_O). Conditioned media were collected and immunoprecipitated with the antibody W02. WB analysis was performed using the antibody W02.
(TIF)

Figure S9 Control experiments using antibody WO2 for Aβ detection. (A) 5 ng, 10 ng, 20 ng and 40 ng of synthetic Aβ40 and Aβ42 peptides, respectively, were loaded and separated on Tris-Tricine gels. Western blot (WB) analysis to detect Aβ was performed with antibody WO2. (B) Human neuroblastoma SH-SY5Y cells were incubated with 2 µM γ-secretase inhibitor X (Calbiochem, Darmstadt, Germany) and the solvent control. Conditioned media were immunoprecipitated with antibody WO2. Immunoprecipitated proteins were loaded and separated on a Tris-Tricine gel and detected via WB analysis using antibody WO2. In presence of γ-secretase inhibitor, Aβ cannot be detected (negative control). (C) Human neuroblastoma SH-SY5Y cells were incubated with 100 µM GM1 and the solvent control. Conditioned media were immunoprecipitated with antibody WO2. Immunoprecipitated proteins were loaded and separated on a Tris-Tricine gel. WB analysis was performed with antibody WO2. GM1 increases Aβ generation (positive control).
(TIF)

Figure S10 Staining of gangliosides separated via TLC using iodine, orcinol and resorcinol. Brain samples and ganglioside standard were separated via TLC as described in the material and method section and stained with iodine, orcinol and resorcinol. TLC1 was first stained with iodine and afterwards with orcinol. TLC2 was first stained with iodine and afterwards with resorcinol. This analysis shows that gangliosides (GM3, GM1, GD3, GD1a, GD1b and GT1b) can be stained with iodine, orcinol and resorcinol.
(TIF)

Figure S11 Determination of GD3S assay unspecificity. As negative control for the GD3S assay we used ganglioside GM2. Compared to the positive control using ganglioside GM3, GM2 was unable to stimulate GD3S. Only a background signal is obtained.
(TIF)

Table S1 Rowdata obtained from quantitative Real-Time PCR experiments. Tables display all ΔCt values, ΔΔCt values and 2^−(ΔΔCt)^ normalized to mRNA actin levels as described in Livak and Schmittgen [72]. At least three independent RNA preparations of at least three different brains or cell culture dishes were analyzed. Figure numbers refer to the original figures in the manuscript.
(DOC)
