# Supplementary material for: Amyloid Precursor Protein (APP) Mediated Regulation of Ganglioside Homeostasis Linking Alzheimer's Disease Pathology with Ganglioside Metabolism
Source: PLoS One. 2012 Mar 28;7(3):e34095. doi: 10.1371/journal.pone.0034095 (PMC3314703; doi:10.1371/journal.pone.0034095)
Supplement: Table S1 — Rowdata obtained from quantitative Real-Time PCR experiments. Tables display all ΔCt values, ΔΔCt values and 2−(ΔΔCt) normalized to mRNA actin levels as described in Livak and Schmittgen [72]. At least three independent RNA preparations of at least three different brains or cell culture dishes were analyzed. Figure numbers refer to the original figures in the manuscript. (DOC) [file pone.0034095.s012.doc]

| **Figure 1 C** |  |  |  |
| --- | --- | --- | --- |
|  | **∆Ct values** | **∆∆Ct values** | **2^-(∆∆Ct)** |
| **MEF PS1r** | 16,223 | -2,260 | 4,790 |
| **MEF PS1/2-/-** | 13,963 |
| **MEF PS1r** | 13,820 | -1,192 | 2,284 |
| **MEF PS1/2-/-** | 12,628 |
| **MEF PS1r** | 15,800 | -1,975 | 3,931 |
| **MEF PS1/2-/-** | 13,825 |

| **Figure 1 E** |  |  |  |
| --- | --- | --- | --- |
|  | **∆Ct values** | **∆∆Ct values** | **2^-(∆∆Ct)** |
| **wt brain** | 5,973 | 0,133 | 0,912 |
| **APP-/- brain** | 6,107 |
| **wt brain** | 5,973 | 0,020 | 0,986 |
| **APP-/- brain** | 5,993 |
| **wt brain** | 5,730 | 0,300 | 0,812 |
| **APP-/- brain** | 6,030 |
| **wt brain** | 6,460 | -0,957 | 1,941 |
| **APP-/- brain** | 5,503 |
| **wt brain** | 6,577 | -1,260 | 2,395 |
| **APP-/- brain** | 5,317 |
| **wt brain** | 6,530 | -1,253 | 2,384 |
| **APP-/- brain** | 5,277 |
| **wt brain** | 6,483 | -0,960 | 1,945 |
| **APP-/- brain** | 5,523 |
| **wt brain** | 6,580 | -1,247 | 2,373 |
| **APP-/- brain** | 5,333 |
| **wt brain** | 6,357 | -1,573 | 2,976 |
| **APP-/- brain** | 4,783 |

| **Figure 3 A** |  |  |  |
| --- | --- | --- | --- |
|  | **∆Ct values** | **∆∆Ct values** | **2^-(∆∆Ct)** |
| **MEF WT** | 16,340 | -4,270 | 19,293 |
| **MEF ∆CT15** | 12,070 |
| **MEF WT** | 15,573 | -3,637 | 12,438 |
| **MEF ∆CT15** | 11,937 |
| **MEF WT** | 15,857 | -3,560 | 11,794 |
| **MEF ∆CT15** | 12,297 |

| **Figure 3 B** |  |  |  |
| --- | --- | --- | --- |
|  | **∆Ct values** | **∆∆Ct values** | **2^-(∆∆Ct)** |
| **wt brain** | 6,357 | -0,767 | 1,701 |
| **APP ∆CT15 brain** | 5,590 |
| **wt brain** | 6,480 | -0,890 | 1,853 |
| **APP ∆CT15 brain** | 5,590 |
| **wt brain** | 6,333 | -0,810 | 1,753 |
| **APP ∆CT15 brain** | 5,523 |
| **wt brain** | 7,093 | -0,703 | 1,628 |
| **APP ∆CT15 brain** | 6,390 |
| **wt brain** | 6,757 | -0,473 | 1,388 |
| **APP ∆CT15 brain** | 6,283 |
| **wt brain** | 6,877 | -0,987 | 1,982 |
| **APP ∆CT15 brain** | 5,890 |
| **wt brain** | 6,860 | -0,707 | 1,632 |
| **APP ∆CT15 brain** | 6,153 |
| **wt brain** | 6,753 | -0,760 | 1,694 |
| **APP ∆CT15 brain** | 5,993 |
| **wt brain** | 6,390 | -0,447 | 1,363 |
| **APP ∆CT15 brain** | 5,943 |

| **Figure 3 C** |  |  |  |
| --- | --- | --- | --- |
|  | **∆Ct values** | **∆∆Ct values** | **2^-(∆∆Ct)** |
| **MEF ∆CT15** | 9,585 | 0,255 | 0,838 |
| **+AICD** | 9,840 |
| **MEF ∆CT15** | 9,500 | 0,300 | 0,812 |
| **+AICD** | 9,800 |
| **MEF ∆CT15** | 8,877 | 0,638 | 0,643 |
| **+AICD** | 9,515 |
| **MEF ∆CT15** | 9,605 | 0,805 | 0,572 |
| **+AICD** | 10,410 |
| **MEF ∆CT15** | 9,635 | 0,155 | 0,898 |
| **+AICD** | 9,790 |
| **MEF ∆CT15** | 9,160 | 0,440 | 0,737 |
| **+AICD** | 9,600 |
| **MEF ∆CT15** | 9,548 | 0,762 | 0,590 |
| **+AICD** | 10,310 |
| **MEF ∆CT15** | 9,345 | 0,440 | 0,737 |
| **+AICD** | 9,785 |
| **MEF ∆CT15** | 9,095 | 0,395 | 0,761 |
| **+AICD** | 9,490 |

| **Figure 3 D** |  |  |  |
| --- | --- | --- | --- |
|  | **∆Ct values** | **∆∆Ct values** | **2^-(∆∆Ct)** |
| **wt** | 11,743 | -1,117 | 2,169 |
| **Fe65 knockdown** | 10,627 |
| **wt** | 11,700 | -1,067 | 2,095 |
| **Fe65 knockdown** | 10,633 |
| **wt** | 11,653 | -0,860 | 1,815 |
| **Fe65 knockdown** | 10,793 |
| **wt** | 11,743 | -0,657 | 1,576 |
| **Fe65 knockdown** | 11,087 |
| **wt** | 11,700 | -0,697 | 1,621 |
| **Fe65 knockdown** | 11,003 |
| **wt** | 11,653 | -0,717 | 1,643 |
| **Fe65 knockdown** | 10,937 |
| **wt** | 11,847 | -1,220 | 2,330 |
| **Fe65 knockdown** | 10,627 |
| **wt** | 11,773 | -1,140 | 2,204 |
| **Fe65 knockdown** | 10,633 |
| **wt** | 11,913 | -1,120 | 2,174 |
| **Fe65 knockdown** | 10,793 |
| **wt** | 11,847 | -0,760 | 1,694 |
| **Fe65 knockdown** | 11,087 |
| **wt** | 11,773 | -0,770 | 1,705 |
| **Fe65 knockdown** | 11,003 |
| **wt** | 11,913 | -0,977 | 1,968 |
| **Fe65 knockdown** | 10,937 |

| **Figure S6 B** |  |  |  |
| --- | --- | --- | --- |
|  | **∆Ct values** | **∆∆Ct values** | **2^-(∆∆Ct)** |
| **control** | 10,953 |  |  |
| **+Aβ40** | 10,903 | -0,050 | 1,035 |
| **+Aβ42** | 10,890 | -0,063 | 1,045 |
| **control** | 10,373 |  |  |
| **+Aβ40** | 9,640 | -0,733 | 1,662 |
| **+Aβ42** | 9,660 | -0,713 | 1,640 |
| **control** | 10,483 |  |  |
| **+Aβ40** | 10,090 | -0,393 | 1,313 |
| **+Aβ42** | 10,290 | -0,193 | 1,143 |
| **control** | 10,517 |  |  |
| **+Aβ40** | 10,457 | -0,060 | 1,042 |
| **+Aβ42** | 11,077 | 0,560 | 0,678 |
| **control** | 10,203 |  |  |
| **+Aβ40** | 9,857 | -0,347 | 1,272 |
| **+Aβ42** | 9,757 | -0,447 | 1,363 |
| **control** | 10,157 |  |  |
| **+Aβ40** | 10,237 | 0,080 | 0,946 |
| **+Aβ42** | 10,747 | 0,590 | 0,664 |
| **control** | 11,237 |  |  |
| **+Aβ40** | 11,010 | -0,227 | 1,170 |
| **+Aβ42** | 10,877 | -0,360 | 1,283 |
| **control** | 10,400 |  |  |
| **+Aβ40** | 10,860 | 0,460 | 0,727 |
| **+Aβ42** | 10,183 | -0,217 | 1,162 |
| **control** | 10,120 |  |  |
| **+Aβ40** | 9,970 | -0,150 | 1,110 |
| **+Aβ42** | 10,017 | -0,103 | 1,074 |
